# Icariside II: Anticancer Potential and Molecular Targets in Solid Cancers

**DOI:** 10.3389/fphar.2021.663776

**Published:** 2021-04-26

**Authors:** Fei Xu, Qiaolan Wu, Lei Li, Jie Gong, Ran Huo, Wenqiang Cui

**Affiliations:** ^1^ Department of Geriatric Medicine, Affiliated Hospital of Shandong University of Traditional Chinese Medicine, Jinan, China; ^2^ First Clinical Medical College, Shandong University of Traditional Chinese Medicine, Jinan, China; ^3^ Affiliated Hospital of Shandong University of Traditional Chinese Medicine, Jinan, China; ^4^ Department of Neurology, Affiliated Hospital of Shandong University of Traditional Chinese Medicine, Jinan, China

**Keywords:** Icariside II, tumor, chemopreventive property, Epimedii, anti-apoptosis

## Abstract

Icariside II, an active flavonoid, is extracted from the traditional Chinese medicinal herb *Epimedii*. It possesses multiple biological and pharmacological properties, including anti-inflammatory, anticancer, and anti-osteoporotic properties. In recent years, apoptosis has become the hot spot in anticancer therapies. Icariside II exerts positive effects on inducing apoptosis and inhibiting proliferation in various cancers. The antitumorigenic activity of Icariside II was also proven through cell cycle arrest, triggering autophagy, reducing cellular metabolism, and inhibiting cancer metastasis and tumor-associated angiogenesis. Additionally, Icariside II, as a natural product, contributed to a synergistic effect alongside chemotherapeutic drugs. Due to its poor aqueous solubility and permeability, more strategies were developed to improve its therapeutic effects. This review aimed to summarize the chemopreventive properties of Icariside II in solid tumors and reveal its underlying molecular mechanisms.

## Introduction

Cancer is the second leading cause of death worldwide, after cardiovascular diseases, and it claimed the lives of 9.6 million individuals in 2018 (Ambekar and Kandasubramanian, 2019). The four most common cancers include lung cancer, colorectal cancer, breast cancer, and prostate cancer. In the past decades, the increasing understanding of cancer biology drove the rapid development of new anticancer therapeutics, including chemotherapy, radiotherapy, surgery, targeted therapy, and immunotherapy. However, despite the rapid expansion in drugs and therapeutic options, there are still few malignancies that respond well to these agents. Moreover, the prognosis of some cancer patients remains very poor, accompanied by recurrence or metastasis. It has, therefore, become necessary to seek novel treatment strategies.

**FIGURE 1:**
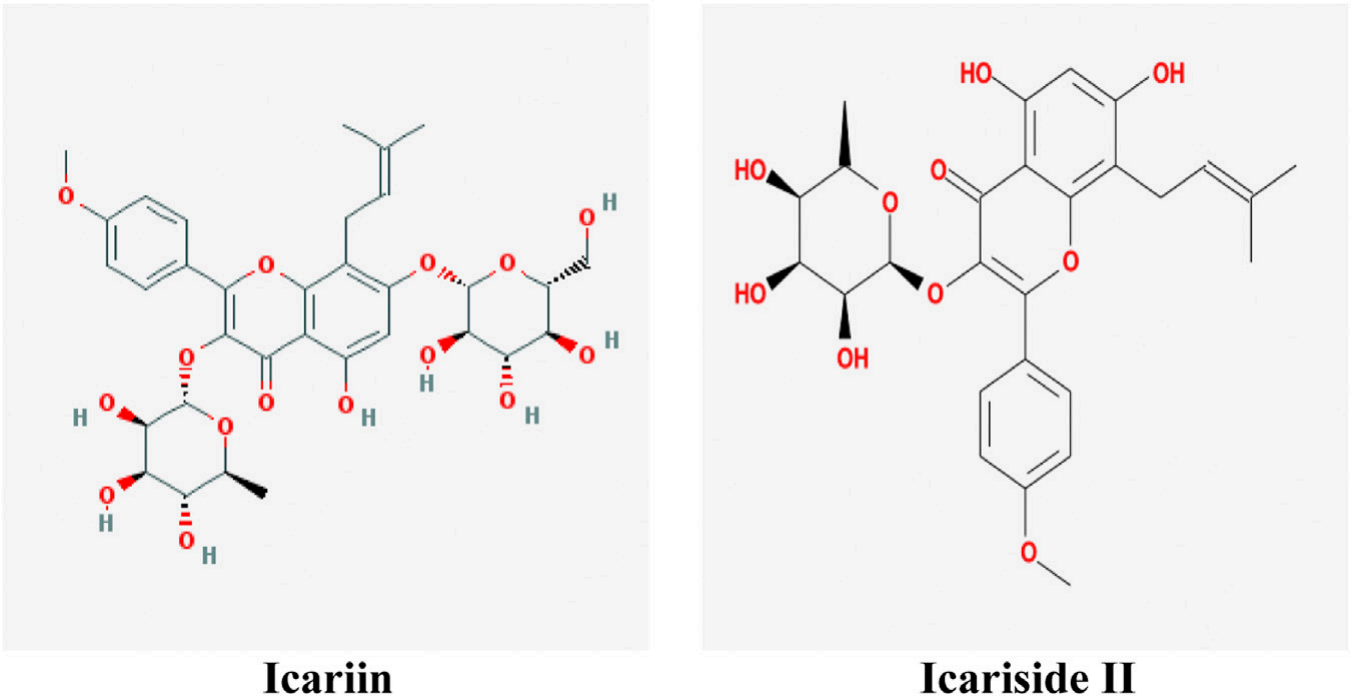
Molecular structure of icariin and Icariside II.

At present, natural products have gradually become acceptable for preventing or treating cancer due to their safety and low toxicity. There are reports showing that almost 80% of anticancer drugs contain natural compounds or mimic their efficacy and construction (Bishayee and Sethi, 2016). *Herba Epimedii* (commonly named as Yin-yang-huo or Xin-ling-pi in China) is commonly used as a traditional herb or functional food to prevent diseases in China. It was first recorded in the Chinese medical classic *Shennong Classic of Materia Medica (*commonly named as *Shen Nong Ben Cao Jing* in China*)* 400 years ago. Icariin (C_33_H_40_O_15_, 676.60 g/mol, Figure 1) is one of the major substances found in *Herba Epimedii*, and Icariside II (also named baohuoside I, C_27_H_30_O_10_, 514.57 g/mol, Figure 1) is the main pharmacological metabolite of icariin *in vivo* and can be obtained from icariin under enzymatic hydrolysis conditions.

Over the past decades, *in vivo* and *in vitro* experimental research into the use of Icariside II in the treatment of various cancers has been performed, showing the positive effects of Icariside II on cancers without systemic toxicity. Its antitumorigenic activity is mediated by induction of apoptosis and autophagy, triggering cancer cell cycle arrest, reducing cellular metabolism, and inhibition of metastasis and tumor-induced angiogenesis. Furthermore, Icariside II supplementation could enhance chemosensitivity and act synergistically and additively to current anticancer drugs, et al.

In this article, we aimed to review the anticancer properties of Icariside II in solid tumors, focusing on its underlying molecular mechanisms.

## Toxicity and Pharmacokinetics of Icariside II

### *In Vitro* Anticancer Pharmacological Properties of Icariside II

#### Apoptosis Induction

Apoptosis is the most common type of programmed cell death, which depends on the activation of caspase proteases. Caspases, the primary mediators of apoptosis, are triggered by two main, interconnected pathways: the extrinsic and intrinsic pathways. The extrinsic pathway, also known as the receptor-mediated pathway, relies on the activation of death receptors (DRs) binding to their proapoptotic ligands on the cell membrane. This is defined as a transmembrane-receptor–mediated interaction in charge of transmitting the death signal from the cell surface to the intracellular area (Lee et al., 2020). The DRs are members of the tumor necrosis factor (TNF) receptor gene superfamily—TNF, FasL, and TNF-related apoptosis–inducing ligand (TRAIL), predominantly produced by immune cells, such as T cells, NK cells, macrophages, and dendritic cells (DC) (Goelz et al., 2021). When the DR is bound to the corresponding death ligand in the cell membrane, it triggers the recruitment of monomeric procaspase-8 and integration of the death-inducing signaling complex (DISC), mainly comprising the FAS-associated death domain (FADD) or TNFR-associated death domain (TRADD). Once the DISC is formed, the autocatalytic processes of procaspase-8 begin. Moreover, the interaction of procaspase-8 with the DISC can also induce myristoylation of cytoplasmic BH3-interacting domain death agonist to promote apoptosis *via* one or the other of two distinct sub-pathways. In contrast, the intrinsic apoptotic pathway, also known as the mitochondrial pathway, is triggered by widespread mitochondrial outer membrane permeabilization (MOMP). This occurs after deadly apoptotic stresses such as DNA damage, cytokine deprivation, endoplasmic reticulum stress, and growth factor withdrawal. It is regulated by proapoptotic and antiapoptotic members of the BCL-2 family of proteins (Galluzzi et al., 2018). According to the number and structure of the BCL-2-homology (BH) domains, BCL-2 family proteins are categorized into three subgroups (BH1-4): the proapoptotic BH3-only proteins (e.g., BIM, NOXA, and BAD), the proapoptotic multidomain proteins (e.g., BAX and BAK), and the antiapoptotic proteins (e.g., BCL-2, BCL-XL, and MCL-1). Meanwhile, the proteins BAX and BAK provoke a release of cytochrome c from the mitochondria into the cytoplasm and subsequently trigger the activation of caspase-9 and downstream caspases such as caspase-3, caspase-6, and caspase-7, ultimately causing apoptosis.

**FIGURE 2:**
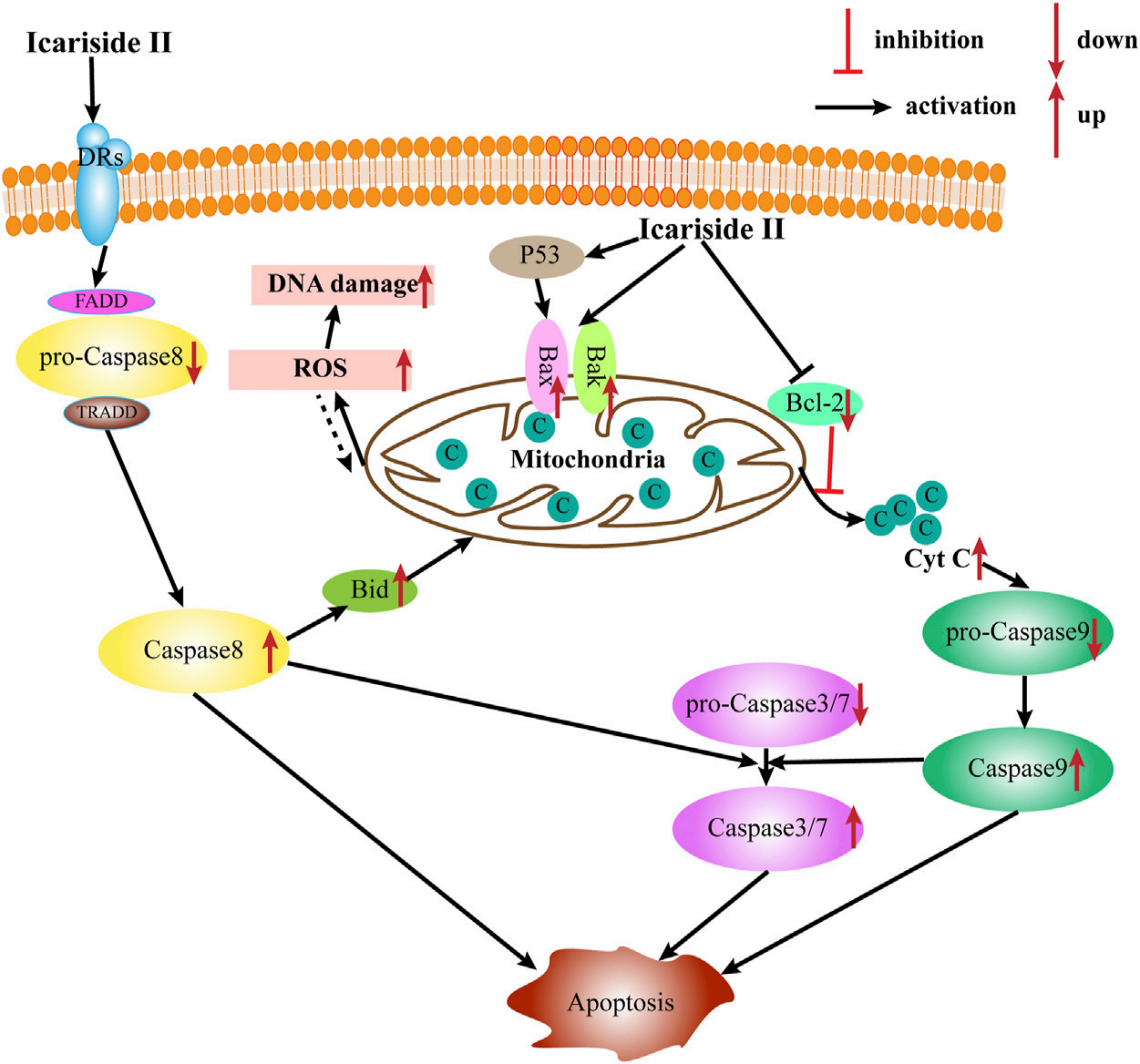
Apoptosis induction by Icariside II intrinsic and extrinsic pathways.

Induction of tumor cell apoptosis is an approach and hot spot of tumor treatment. There is much evidence indicating that Icariside II exerts apoptosis-inducing effects in various cancer cell lines (Table 1; Figure 2). After exposure to Icariside II, the expression of cleavage of caspase-3/8/9/7/PARP and Bax increases in concentration- and time-dependent manners, but the antiapoptotic factor BCL-2 was decreased in human liver cancer cell lines (Guo et al., 2020a). In prostate cancer, Icariside II was shown to activate caspase-3, promote cytochrome c release into cytosol, and simultaneously reduce the expression of the inactive, proenzymatic form of caspase-9 (Lee et al., 2009). The apoptotic mechanism in human glioma cells is activated by elevating the cleavage of caspase-3/8, BCL-2-associated protein (Bax), cytochrome c, and reducing the BCL-2 level (Quan et al., 2017; Guo et al., 2020b). Human melanoma cells displayed morphological changes indicative of the early apoptotic stage when treated with Icariside II and a remarkable elevation was seen in the expression of the cleavage of caspase-3 and PPAR (Wu et al., 2012; Peng and Zhang, 2018). Another study also showed that the ratio of Bax to BCL-2 proteins increased in a concentration- and time-dependent manner in pancreatic cancer (Ni et al., 2019). Its proapoptotic effect was also demonstrated in breast cancer and non-melanoma skin cancer, where Icariside II induced the release of cytochrome c through the mitochondrial membrane to the cytosol, subsequently leading to the activation of caspase-3/7/9 and PARP cascade (Huang et al., 2012; Wu et al., 2013). In a recent study, Icariside II was shown to inhibit the growth of cervical cancer HeLa cells and induce apoptosis through improving caspase-3/9 activity, p53, cytochrome c expression, and modulate the BCL-2 family of proteins (Sun et al., 2020a). Altogether, a body of evidence supports the idea that Icariside II has powerful proapoptotic activity through the intrinsic apoptosis pathway, and there is no data indicating the influence of Icariside II on apoptosis mediated by the extrinsic pathway.

**TABLE 1:** Anticancer effects of Icariside II *in vitro*.

Cell lines used		Concentration tested	Efficacy, IC50 (exposure time)	Anticancer effects	Molecular targets	References
Human liver cancer cell	HuH-7, HepG2	20, 50 μM	HuH-7: 32 μM (24 h)	Proapoptosis and inhibition of proliferation, colony formation, invasion, and migration	Ki67↓, MMP2/9↓, cleaved caspase-3/9↑, Bax/BCL-2↑, p-mTOR↓, p-S6K1↓, p-AMPKα1↑	Guo et al. (2020b)
			HepG2: 34 μM (24 h)			
	HepG2	20, 25, 30 μM	Unclear	Proapoptosis and induction of autophagy	Mitochondrial membrane potentials↓, Bax/BCL-2↑, cleaved Bid↑, cleaved caspase-3/9/8/7/PPAR↑, LC3B↑, LAMP1↑, SQSTM1↑, lysosome dysfunction↑, autophagosome degradation↓, autophagosome accumulation↑	Geng et al. (2015)
Human prostate cancer	PC-3	10, 20, 40 μm	20 μM (24 h)	Proapoptosis and arrest of cell cycle	Mitochondrial membrane potentials↓, Cyct C↑, cleaved caspase-3/PPAR↑, caspase-9↓, COX-2↓, iNOS↓, VEGF↓	Lee et al. (2009)
	DU145	20, 40 μM	Unclear	Proapoptosis, arrest of cell cycle, inhibition of invasion and migration, and induction of autophagy	LC3-II/I↑, Beclin-1↑, PI3K↓, p-Akt↓, p-mTOR↓	Li et al. (2020)
Human glioma cells	U251	20, 50 μM	Unclear	Proapoptosis and inhibition of proliferation, invasion, and migration	Ki67↓, Bax↑, BCL-2↓ cleaved caspase-3/9↑, JNK and NF-κB activities↑, p-S6K1↓, p-AMPKα1↑, mTOR activities↓	Guo et al. (2020a)
	U87, A172	20, 40 μM	Unclear	Proapoptosis, arrest of cell cycle, and inhibition of proliferation, migration, and invasion	Cleaved caspase-3/PPAR↑, Cyclin D↓, p53↑, Cyct C↑, p21↑, p27↑, Akt activities↓, FOXO3a activity↑	Quan et al. (2017)
Melanoma cells	M14, MV3	20 μg/ml	Unclear	Inhibition of proliferation and migration	miR-144↑	Peng and Zhang (2018)
	A375	25, 50, 100 μM	Unclear	Proapoptosis, inhibition of proliferation, and arrest of cell cycle	CDK2↓, cyclin E↓, P-CDK1↓ and cyclin B1↓, ROS↑, p-p53↑, p-p38↑, p21↑, cleaved PPAR↑	Wu et al. (2015)
Human pancreatic cancer cells	PANC-1, CFPAC-1	25, 50 μM	Unclear	Proapoptosis and inhibition of proliferation, migration, and invasion	Mitochondrial	Ni et al. (2019)
					Respiration↓, glycolysis↓, p62↓, LC3-II/I↑, Bax↑, BCL-2↓, Cleaved caspase-3/8↑, p-S6K1↓, p-AMPKα1↑, mTOR activities↓	
Human breast cancer cells	MCF7	25, 50, 75 μM	50 μM (72 h)	Proapoptosis	Cleaved caspase-3/7/9/8/PPAR↑, caspase-3/7/9/8↓, PPAR↓, mitochondrial membrane potential↓, cytosol cyto C/AIF↑, mitochondrial cyto C/AIF↓, Fas↑, FasL↓, FADD↑, Bax↑, Bcl-xL↑, BimL↑	Huang et al. (2012)
	MDA-MB-231	6.25, 12.5, 25 μM	Unclear	Inhibition of invasion	CXCR4↓, NF-κB↓	Kim and Park (2014)
Human epidermoid carcinoma cells	A431	10, 25, 50 μM	Unclear	Proapoptosis	Cleaved caspase-9/PPAR↑, caspase 9↓, PPAR↓, AKT/STAT3/ERK activities↓, EGFR activities↓	
Human cervical cancer cells	Hela	10, 20, 30 μM	9.2 μM (48 h)	Arrest of cell cycle, inhibition of proliferation, and proapoptosis	p-AKT↓, cyclin D↓, CDK 6↓, CDK 4↓, cyclin A↓, cyclin E↓, CDK 2↓, ROS↑, Fas↑, TNF-R1↑, caspase 8/3/9↓, Bax↑, BCL-2↓, P53↑, Bak↑, cyct C↑, mitochondrial membrane potential↓	Sun et al. (2020b)
	Hela	5, 10, 20, 30 μM	Unclear	Inhibition of invasion and migration	MMP2/9↓, JNK and NF-κB activities↑	Sun et al. (2020a)
	Hela	6.25, 12.5, 25 μM	Unclear	Inhibition of invasion	CXCR4↓, NF-κB↓	Kim and Park (2014)
Human NSCLC cells	A549	6.25, 12.5, 25 μM	25.1 μM (24 h)	Proapoptosis	ROS↑, mitochondrial membrane potential↓, Bax/BCL-2↑, cleaved caspase-3/9/PPAR↑, caspase-3/9↑, p-p38↑, p-JNK↑	Song et al. (2012)
			11.5 μM (48 h)			
			9.6 μM (72 h)			
Human esophageal cancer cell	Eca109	12.5 μg/ml, 25 μg/ml, 50 μg/ml	24.8 μg/ml (48 h)	Proapoptosis and arrest of cell cycle	Survivin↓, Cyclin D1↓, β-catenin↓	Wang et al. (2011)
Human osteosarcoma cells	MG-63, Saos-2	20, 25, 30 μM	Unclear	Inhibition of proliferation	EGFR/mTOR activities↓	Geng et al. (2014)
	HOS	0.1, 1, 10 μM	Unclear	Suppression of cell growth and inhibition of angiogenesis, migration, invasion, and glucose metabolism	HIF-1α↓, VEGF↓, uPAR↓, ADM↓, MMP2↓, aldolase A↓, and enolase 1↓	Choi et al. (2008)

Increased reactive oxygen species (ROS) production is detected when oxidative stress occurs. Although ROS are mainly generated in mitochondria, they can cause oxidative damage to mitochondrial DNA, proteins, and lipids, which affect the BCL-2 family of proteins and cytochrome c, ultimately triggering apoptosis *via* the intrinsic and extrinsic pathways (Groeger et al., 2009). Icariside II could induce apoptosis *via* the overproduction of ROS, while NAC, a scavenger of ROS, diminished the effect in human non–small-cell lung cancer (NSCLC) (Song et al., 2012).

#### Cell Cycle Arrest

The cell cycle consists of four sequential phases: the G1, S, G2, and M phases. The first step is called the G1 phase, and it is vital for cell proliferation and sensitive to growth signaling networks; the S phase is a time for DNA synthesis and its complete duplication; G2, the gap phase, is when the cell prepares for entry into mitosis; the M phase is when the cell divides into two genomically stable daughter cells (Mansilla et al., 2020). The cell cycle is tightly regulated by cyclin-dependent kinases (CDKs) that coordinate with their cyclin partners (Mansilla et al., 2020). Uncontrolled CDK activity is frequently seen in cancer cells and cyclin-dependent kinase inhibitors (CKIs) possess antitumor properties by restricting the kinase activity. Therefore, inhibition of CDK activity is an effective strategy to inhibit cancer.

**FIGURE 3:**
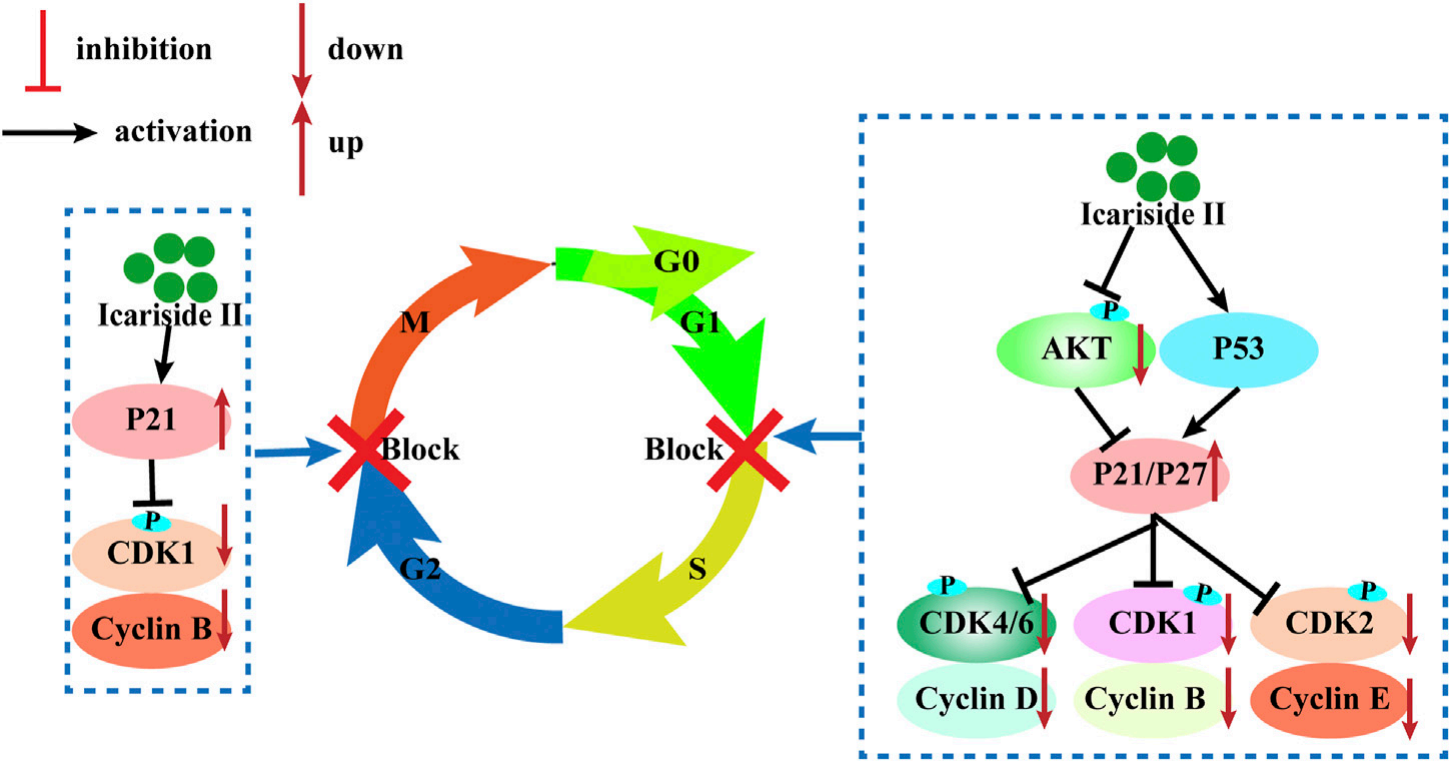
Cell cycle arrest induced by Icariside II.

Icariside II has been investigated for inducing cell cycle arrest (Figure 3). In human glioblastoma cells, Icariside II induced G0/G1 cell cycle arrest by downregulating G1/S transition cyclin D whilst upregulating cyclin-dependent kinase inhibitors p21 and p27, dependent on the Akt-FOXO3a pathway. Likewise, Icariside II increased the percentage of A375 human melanoma cells at the G0/G1 boundary with downregulations of cyclin E, cyclin B1, CDK2, and phosphorylated-CDK1 (p-CDK1) (Wu et al., 2015). Besides the inhibition of G1/S phase cell cycle regulatory proteins, Icariside II could also trigger cell cycle arrest through the generation of ROS by activating the 3 M MAPK and p53 signaling pathways (Wu et al., 2015). In human esophageal squamous carcinoma Eca109 cells, Icariside II was demonstrated to be highly effective in downregulating β-catenin and cyclin D1 protein expression and stochastically inducing cell cycle arrest (Wang et al., 2011). The cell cycle blockage in human PC-3 and DU145 prostate cancer cells at the G0/G1 checkpoint was caused by Icariside II in a dose-dependent manner, which increased the percentage of cells undergoing G1 phase cell cycle arrest to 45.7–80% upon treatment with 40 µM (Lee et al., 2009; Li et al., 2020). These results were supported in a study by Wei Z, which showed that Icariside II induced significant accumulation of cells at the G1/G0 phase with decreased cell populations at the S phase and the G2/M phase in cervical cancer (Sun et al., 2020). This is attributed to the downregulation of p-AKT, CDK2, and cyclin E but no changes in cyclin D, CDK6, and cyclin A expressions (Sun et al., 2020a). These studies have presented evidence that Icariside II–induced cell cycle arrest of cancer cells predominantly occurs during the G0/G1 phase by modulating the expressions and kinase activities of cyclin, CDKs, p21, p53, and other signaling pathways.

#### Autophagy Triggering

Autophagy is a physiological cellular process, whereby misfolded proteins, damaged or aged organelles, and mutated proteins are degraded, fused with lysosomes, and generate the autolysosome (Behrends et al., 2010). It is hallmarked by a lysosome-dependent process and predominately regulated by autophagy-related genes (ATG). The process is divided into four steps in a given order: initiation, nucleation, maturation, and degradation, all of which are controlled by different ATG proteins (Mercer et al., 2018). Several molecules are involved in autophagic processes; these include mammalian target of rapamycin (mTOR), AMP-activated protein kinase (AMPK), Beclin-1, autophagy-related proteins and microtubule-associated protein 1 light chain 3 (LC3), etc. (Mercer et al., 2018). Autophagy has dichotomous roles in tumor promotion and suppression. In the early stages of cancer progression, autophagy acts as a tumor suppressor by removing damaged proteins and organelles, inhibiting inflammation, limiting genomic instability, inducing stress, and suppressing ROS production by the mitochondria (Lim and Murthy, 2020; Yun et al., 2020). In contrast, when tumors are already established, autophagy promotes cancer cell survival and growth by reprogramming metabolism, promoting angiogenesis, and promoting hypoxia and oxidative stress (Das et al., 2018; Thomas et al., 2018). Given that autophagy plays a role in enhancing tumor cell death, initiation of autophagy may be an attractive strategy for anticancer therapies.

Icariside II, as a novel phosphodiesterase 5 inhibitor, was found to play a role in regulating autophagy *via* the PI3K/AKT/mTOR, ROS/GSK-3β/mitochondrial, and PKG/GSK-3β signaling pathways in different diseases (Gao et al., 2017; Gao et al., 2020; Li et al., 2020; Zhang et al., 2020). In human prostate cancer, autophagy induced by Icariside II was characterized by enhanced autophagosome formation, reduction in P70S6K expression, and upregulation of LC3 and Beclin-1 by inhibiting PI3K-AKT-mTOR activity (Li et al., 2020). Till date, the influence of Icariside II on autophagy in different cancer cell lines is not well elucidated and more studies are needed to shed better light on this subject.

#### Inhibition of Cancer Cell Metastasis

Metastasis is the leading cause of morbidity and mortality in cancer patients (Kudo-Saito et al., 2021). It is a multistep process through which cancer cells spread from the primary tumor to the bloodstream, lymphatic system, or distant parts of the body. It involves four steps: detachment, migration, invasion, and formation. In most cases, there is no effective therapy for metastatic cancer, and metastasis is regarded as a predictor of the worst prognosis in cancer development. Hence, inhibition of metastasis is an enormous challenge for clinicians and of vital importance to control cancer and prolong patients’ overall survival. Several molecular mechanisms are closely correlated with metastasis, such as epithelial–mesenchymal transition (EMT), angiogenesis, and extracellular matrix (ECM) degradation (Majidpoor and Mortezaee, 2021). EMT plays an especially key role in metastasis of cancer cells. Matrix metalloproteinases (MMPs), as zinc-dependent endopeptidases, are able to catalyze the proteolytic activities and induce degradation of the ECM. Specifically, MMP-2 and MMP-9, whose catalytic domains are unique to fibronectin type II, are necessary for gelatin digestion and favor tumorigenic processes. Compared with nonmalignant tissue, the levels of MMP-2 and MMP-9 expression are higher in tumors (Scheau et al., 2019; Majidpoor and Mortezaee, 2021). Moreover, some chemokines and their receptors are reported to be closely linked to the metastasis of cancers. These include CXC chemokine ligand (CXCL) 12 and its receptor, CXCR4 (Yang et al., 2015; Nguyen et al., 2020). The level of CXCL12 is higher in metastatic sites (Balkwill, 2004).

Accumulating evidence is indicating that Icariside II has the ability to control cancer cell invasion and migration in lung cancer (Song et al., 2017), hepatocellular carcinoma (Guo et al., 2020a), cervical cancer (Sun et al., 2020b), osteosarcoma (Choi et al., 2008), melanoma (Peng and Zhang, 2018), and prostate cancer (Li et al., 2020). In hepatocellular carcinoma (Guo et al., 2020b) and cervical cancer (Sun et al., 2020a), it has been demonstrated that Icariside II could weaken the migratory and invasive ability of HuH-7, HepG2, and HeLa cells through inhibiting metastasis-related protein MMP2/9 expression. CXCR4 is expressed on human cervical cancer HeLa cells and breast cancer MDA-MB-231 cells. Under the conditions of Icariside II treatment, this expression of CXCR4 is suppressed by inhibiting NF-κB activation (Kim and Park, 2014). In addition, the MAPK signaling pathway, including P38 MAPK, ERK, and JNK, participated in Icariside II regulation (Sun et al., 2020b). It is a well-established fact that an active inflammatory microenvironment created by immune cells and inflammatory cytokines is favorable for cancer invasion and metastasis by modulating EMT (Ma et al., 2016; Yan et al., 2018). Similarly, in lung cancer, the results of wound healing and the transwell assay indicated that IL-6 and TNF-α contribute remarkably to wound closure and cell invasion. These effects were partially reversed by Icariside II through upregulating E-cadherin and downregulating N-cadherin and Vimentin *via* inactivation of the Akt/NF-kB/GSK-3β pathway (Song et al., 2017). Apart from these, in a study with human osteosarcoma cells, Icariside II suppressed cancer cell metastasis by decreasing the protein level of HIF-1α and simultaneously inducing a decrease in HIF-inducible genes, which are closely associated with angiogenesis and glucose metabolism (Choi et al., 2008). The molecular mechanisms used by Icariside II involved the inhibition of the vascular endothelial growth factor (VEGF), the urokinase plasminogen activator receptor (uPAR), adrenomedullin (ADM), MMP2, aldolase A, and enolase 1 (Choi et al., 2008).

#### Inhibition of Angiogenesis

Blood vessel walls are composed of endothelial cells (ECs) and mural cells. Tumor-associated vessels are significantly different from physiological ones and are characterized by rapid formation, disorganized structure, and high permeability (Ribatti et al., 2000). New vessels begin to form when a tumor grows; they provide cancer cells with nutrients, oxygen, and growth factors. Moreover, cancer-associated vessels regulate the entrance and egress of circulating leukocytes or other immune cells through chemokines and other molecules on the endothelial cell surface (Baeriswyl and Christofori, 2009). Thus, angiogenesis plays a crucial role in tumor growth, invasion, migration, and tumor immune surveillance. The VEGF is deemed a pivotal mediator that stimulates the pro-angiogenic activity; thus, anti-angiogenic therapy, especially anti-VEGF or VEGF antagonist therapy, is a common strategy for combined therapy in cancer treatment (Teleanu et al., 2019; Guo and Cui, 2020). Tumor-hypoxia contributes to angiogenesis *via* upregulating VEGF and VEGF receptors. Concerning HOS osteosarcoma cells, Icariside II attenuated the positive effect of HIF on VEGF expression (Choi et al., 2008). The tube formation assay showed that Icariside II broke the endothelial network-like structures induced by HIF (Choi et al., 2008). It is necessary to go one step further to explore the anti-angiogenic properties of Icariside II in cancers.

#### Adjutant Therapy

Even though chemotherapy, combined with radiotherapy, targeted therapy, and immunotherapy, is a conventional therapy for many cancers, the clinical outcomes remain unsatisfactory, with issues such as drug-induced multi-drug resistance (MDR), serious adverse effects, and poor therapeutic response (Burrell et al., 2013). Combination therapy is considered a good therapeutic strategy for improving therapeutic efficacy and tackling MDR. Effort has been put into finding non-chemotherapeutic drugs, such as natural products, for improving anticancer efficacy and outcomes of combination therapy and, to some extent, reducing toxic side effects.

Icariside II, as a kind of natural product, has not only exhibited antitumor activities applied as a single agent but also contributes to a synergistic effect with chemotherapeutic drugs for malignant melanoma (Wu et al., 2012; Table 2). Paclitaxel is one of the most important cancer chemotherapeutic drugs and is widely used for the treatment of various cancers. The TLR4 signal, combined with its adaptor protein MyD88, could activate its downstream ERK, P38, or JNK and then promote tumor growth, metastasis, and chemoresistance (Guha et al., 2001). MyD88 is a potent biomarker for predicting paclitaxel response (Silasi et al., 2006). Icariside II treatment leads to the improvement of the paclitaxel-induced apoptosis by inhibiting the TLR4/MyD88/ERK signaling pathway in human melanoma A375 cells (Wu et al., 2012). The tumor necrosis factor–related apoptosis-inducing ligand (TRAIL/Apo2L) is a promising agent for triggering apoptosis while some cancer cells with overexpression of FLICE-like inhibitory protein (cFLIP) are resistant to it (Kataoka, 2005). Icariside II can reverse the drug resistance in A375 melanoma cells by downregulating the expressions of cFLIP mediated by the ROS-STAT3 or NF-κB signaling pathways (Du et al., 2016).

**TABLE 2:** Efficacy of Icariside II in combinational cancer therapy.

Cell lines used		Concentration used (μM)	Drugs	Effects	Molecular targets	References
Human melanoma cells	A375	10	Paclitaxel	Proapoptosis	Cleaved caspase-3↑, VEGF↓, IL-8↓,TLR4↓, MyD88↓, p-ERK↓	Wu et al. (2012)
Human melanoma cells	A375	20	Recombinant TRAIL	Proapoptosis	ROS↑, caspase-3/8/9↓, PARP↓, cFLIP↓, STAT3/AKT activity↓, NF-κB activity↓	Du et al. (2016)

### *In Vivo* Therapeutic Potential of Icariside II

There is much evidence indicating that Icariside II inhibits growth, invasion, and migration and prevents drug resistance in cancer cells *in vitro*. The anticancer potential of Icariside II has also been demonstrated in *in vivo* investigations. Nude mice xenografts, established using HCC cells, were treated with 25 mg/kgd Icariside II for 30 days and exhibited a remarkable reduction in tumor volume, weight, and the protein levels of MMP2/9 and BCL-2/Bax ratio compared to the DMSO-treated group (Guo et al., 2020a). The antitumor effects of Icariside II on reducing tumor weight and volume have been verified in other animal models such as HeLa-bearing mice (Sun et al., 2020a), sarcoma-180 tumor ICR mice (Geng et al., 2014), M14 xenograft B-NSG nude mice (Peng and Zhang, 2018), a U251 human xenograft model (Guo et al., 2020b), BALB/c mice with four T^1^-Neu cells (Zhou et al., 2011), and an Eca109 xenograft model (Wang et al., 2011). In these reports, the anticancer efficiency of Icariside II is achieved through inhibiting cellular proliferation (Geng et al., 2014), enhancing cell apoptosis (Guo et al., 2020a), blocking the cell cycle (Wang et al., 2011), and modifying the inflammatory microenvironment (Zhou et al., 2011). The detailed information is presented in Table 3. Moreover, Icariside II not only inhibits tumor growth and metastasis but also causes no adverse effects on the body; this was confirmed by a study (Sun et al., 2020b) in which the body weight and main organ weight showed no changes.

**TABLE 3:** Therapeutic potential of Icariside II *in vivo*.

Cancer type	Animal subject	Model	Dose	Days	Route of administration	Results and mechanisms of action	Reference
Live	Male BALB/c nude mice	Subcutaneously	25 mg/kg·d	30	Intragastric administration	Reduction of tumor volume and tumor weight and downregulation of MMP-2, MMP9, BCL-2, and p-mTOR while upregulation of bax expressions	Guo et al. (2020b)
Cervical	Female BALB/c nude mice	Subcutaneously	25 mg/kg·d	12	Vein injection	Reduction of tumor volume and weight by inducing cell apoptosis and downregulation of MMP2/9	Sun et al. (2020a); Sun et al. (2020b)
Osteosarcoma	Male ICR mice	Subcutaneously	10, 20, and 30 mg/kg·d	10	Intraperitoneal injection	Inhibition of cell proliferation via the EGFR/mTOR signaling pathway and downregulation	Geng et al. (2014)
						of Ki-67 expression	
Melanoma	Female B-NSG nude mice	Subcutaneously	25 mg/kg, every two days	20	Vein injection	Reduction of tumor size and weight	Peng and Zhang (2018)
Glioma	Male BALB/c nude mice	Subcutaneously	35 mg/kg·d	30	Intraperitoneal injection	Inhibition of tumor volume and tumor weight through the mTOR/AMPKα signal pathway	Guo et al. (2020a)
Breast	Female BALB/c mice	Subcutaneously	100 mg/kg, three times a week	28	Intraperitoneal injection	Reduction of tumor volume and decreasing number of Gr-1^+^CD11b^+^ MDSCs in the spleen	Zhou et al. (2011)
Esophagus	Female BALB/c nude mice	Subcutaneously	25 mg/kg, every two days	21	Intralesional injection	Reduction of tumor size	Wang et al. (2011)

### Approaches to Strengthen Icariside II Absorption and Delivery

Animal and cell experiments have provided evidence that Icariside II is a potential chemopreventive agent. Unfortunately, Icariside II possesses low oral absorption due to its poor aqueous solubility, permeability, and efflux, which restricts its clinical application. Therefore, it is essential to develop a formulation for Icariside II to increase its solubility and absorption and improve its therapeutic effects (Table 4).

**TABLE 4:** Application of drug delivery systems in Icariside II.

	Model	Results	Reference
Phospholipid	Caco-2 cell monolayer; male rats	Absorptive permeability, secretory permeability↑, efflux ratio↓, maximum concentration↑, time to maximum concentration↓, relative bioavailability↑	Jin et al. (2012)
Phospholipid + TPGS	Caco-2 cell monolayer; male rats	Solubility↑, instantaneous release↑, permeability↑, efflux ratios↓, maximum concentration↑, time to maximum concentration↓, relative bioavailability↑	Jin et al. (2013)
TPGS + Solutol HS 15	A549 cells	Solubility↑, cellular uptake↑, half-maximal inhibitory concentration↓, toxic effects↓, antitumor efficiency↑	Yan et al. (2016)
Solutol HS15 + Pluronic F127	Caco-2 cell monolayer; male rats	Solubility↑, instantaneous release↑, cellular uptake↑, efflux ratios↓, relative bioavailability↑, no apparent toxicity (-)	Jin et al. (2012)

Phospholipids are an important component of the cell membrane and promote drug absorption. Phospholipid complex has been used for resolving oral bioavailability–related issues. A study showed that Icariside II-phospholipid complex (a nanoscale size, 81 ± 10 nm) resulted in a significant elevation of absorptive permeability and a dramatic decrease in efflux ratio *in vitro* and a higher relative bioavailability *in vivo* (Jin et al., 2012)*.* The maximum concentration and time to maximum concentration were significantly higher in the baohuoside I group (296.32 ng/ml *vs*. 525.37 ng/m, 50 *vs*. 52.5 min) (Jin et al., 2012). Even though phospholipid complexes made obvious contributions to gastrointestinal absorption, their solubility is low which also limits their application. D-α-tocopheryl polyethylene glycol 1,000 succinate (Vitamin E TPGS), or TPGS, is a water-soluble derivative of natural vitamin E and has been extensively used as a carrier for enhancing drug solubility, permeability, and a sustained, controlled, and targeted drug delivery (Shah and Banerjee, 2011). The data collected by Xiao-Bin Jia *et al.* showed that TPGS/Icariside II-phospholipid complex (at a ratio of 9:1) appears to result in a faster absorption rate, higher plasma concentration, longer circulation time, an increase in cellular uptake of drugs, and reduction of efflux *in vitro* and *in vivo* (Jin et al., 2013)*.* Another way to enhance the anticancer activities is to pack Icariside II with TPGS and Solutol HS 15 (BTSM). Solutol HS 15 is a novel amphiphilic surfactant with many advantages, such as high solubility capacity, biocompatibility, and performance and low adverse effects (Yan et al., 2016). Icariside II in the presence of BTSM is more effective than Icariside II alone in inhibiting NSCLC cell proliferation and reducing tumor weight and volume in the NSCLC mice model (Yan et al., 2016). Similar results were obtained in mixed micelles, in which solutol HS15 and pluronic F127 were used as surfactants (Hou et al., 2016). Its solubility peaked to 11.7 mg/ml, increasing up to 900 fold while the efflux ratio decreased by 83.5% in Caco-2 cell culture models (Hou et al., 2016). In addition, BTSM demonstrates the properties of increasing the relative bioavailability, which increased about 3-fold (4,059.81 mg/l·h *vs*. 12,886.76 mg/l·h) with gastrointestinal safety in rats (Hou et al., 2016).

## Conclusion

Malignancy is a global health problem that is threatening people’s lives. Even though new therapeutic approaches have been developed and introduced for cancer treatment, the overall cure rate remains unsatisfactory, and adverse events are a critical obstacle to clinical application. Therefore, there is still a need for new drugs and treatment strategies against cancer. Icariside II has been found to have positive effects on multiple types of cancers in numerous studies. In addition, Icariside II exerts synergistic effects when combined with chemotherapeutic drugs, such as paclitaxel, and other agents, such as TRAIL. However, it is not clear whether Icariside II has the potential to reduce the adverse effects of other therapies. Therefore, further *in vivo* and *in vitro* studies are required to assess the efficacy and safety of Icariside II for chemoprevention and treatment of cancers.

## References

[B1] Ambekar R. S. Kandasubramanian B. (2019). A polydopamine-based platform for anti-cancer drug delivery. Biomater. Sci. 7 (5), 1776–1793. 10.1039/c8bm01642a 30838354

[B2] Baeriswyl V. Christofori G. (2009). The angiogenic switch in carcinogenesis. Semin. Cancer Biol. 19 (5), 329–337. 10.1016/j.semcancer.2009.05.003 19482086

[B3] Balkwill F. (2004). The significance of cancer cell expression of the chemokine receptor CXCR4. Semin. Cancer Biol. 14 (3), 171–179. 10.1016/j.semcancer.2003.10.003 15246052

[B4] Behrends C. Sowa M. E. Gygi S. P. Harper J. W. (2010). Network organization of the human autophagy system. Nature 466 (7302), 68–76. 10.1038/nature09204 20562859 PMC2901998

[B5] Bishayee A. Sethi G. (2016). Bioactive natural products in cancer prevention and therapy: progress and promise. Semin. Cancer Biol. 40-41, 1–3. 10.1016/j.semcancer.2016.08.006 27565447

[B6] Burrell R. A. McGranahan N. Bartek J. Swanton C. (2013). The causes and consequences of genetic heterogeneity in cancer evolution. Nature 501 (7467), 338–345. 10.1038/nature12625 24048066

[B7] Choi H. J. Eun J. S. Kim D. K. Li R. H. Shin T. Y. Park H. (2008). Icariside II from Epimedium koreanum inhibits hypoxia-inducible factor-1α in human osteosarcoma cells. Eur. J. Pharmacol. 579 (1-3), 58–65. 10.1016/j.ejphar.2007.10.010 17980359

[B8] Das C. K. Mandal M. Kögel D. (2018). Pro-survival autophagy and cancer cell resistance to therapy. Cancer Metastasis Rev. 37 (4), 749–766. 10.1007/s10555-018-9727-z 29536228

[B9] Du J. Wu J. Fu X. Kai-Wing Tse A. Li T. Su T. (2016). Icariside II overcomes TRAIL resistance of melanoma cells through ROS-mediated downregulation of STAT3/cFLIP signaling. Oncotarget 7 (32), 52218–52229. 10.18632/oncotarget.10582 27418138 PMC5239546

[B10] Galluzzi L. Vitale I. Aaronson S. A. Abrams J. M. Adam D. Agostinis P. (2018). Molecular mechanisms of cell death: recommendations of the nomenclature committee on cell death 2018. Cell Death Differ 25 (3), 486–541. 10.1038/s41418-017-0012-410.1038/s41418-018-0102-y 29362479 PMC5864239

[B11] Gao J. Deng Y. Yin C. Liu Y. Zhang W. Shi J. (2017). Icariside II , a novel phosphodiesterase 5 inhibitor, protects against H 2 O 2 ‐induced PC 12 cells death by inhibiting mitochondria‐mediated autophagy. J. Cell. Mol. Med. 21 (2), 375–386. 10.1111/jcmm.12971 27642051 PMC5264130

[B12] Gao J. Long L. Xu F. Feng L. Liu Y. Shi J. (2020). Icariside II, a phosphodiesterase 5 inhibitor, attenuates cerebral ischaemia/reperfusion injury by inhibiting glycogen synthase kinase‐3β‐mediated activation of autophagy. Br. J. Pharmacol. 177 (6), 1434–1452. 10.1111/bph.14912 31658364 PMC7056470

[B13] Geng Y. D. Yang L. Zhang C. Kong L. Y. (2014). Blockade of epidermal growth factor receptor/mammalian target of rapamycin pathway by Icariside II results in reduced cell proliferation of osteosarcoma cells. Food Chem. Toxicol. 73, 7–16. 10.1016/j.fct.2014.08.002 25119583

[B14] Geng Y. D. Zhang C. Shi Y. M. Xia Y. Z. Guo C. Yang L. (2015). Icariside II-induced mitochondrion and lysosome mediated apoptosis is counterbalanced by an autophagic salvage response in hepatoblastoma. Cancer Lett. 366 (1), 19–31. 10.1016/j.canlet.2015.05.032 26118776

[B15] Goelz N. Eekels J. J. M. Pantic M. Kamber C. T. Speer O. Franzoso F. D. (2021). Platelets express adaptor proteins of the extrinsic apoptosis pathway and can activate caspase-8. PLoS One 16 (1), e0244848. 10.1371/journal.pone.0244848 33428668 PMC7799768

[B16] Groeger G. Quiney C. Cotter T. G. (2009). Hydrogen peroxide as a cell-survival signaling molecule. Antioxid. Redox Signaling 11 (11), 2655–2671. 10.1089/ars.2009.2728 19558209

[B17] Guha M. O'Connell M. A. Pawlinski R. Hollis A. McGovern P. Yan S.-F. (2001). Lipopolysaccharide activation of the MEK-ERK1/2 pathway in human monocytic cells mediates tissue factor and tumor necrosis factor α expression by inducing Elk-1 phosphorylation and Egr-1 expression. Blood 98 (5), 1429–1439. 10.1182/blood.v98.5.1429 11520792

[B18] Guo F. Cui J. (2020). Anti-angiogenesis: opening a new window for immunotherapy. Life Sci. 258, 118163. 10.1016/j.lfs.2020.118163 32738363

[B19] Guo Y. Wang C. Jiang M. Zhu H. Weng M. Sun L. (2020a). Baohuoside I via mTOR apoptotic signaling to inhibit glioma cell growth. Cancer Manag. Res. Vol. 12, 11435–11444. 10.2147/CMAR.S265803 PMC7667174 33204156

[B20] Guo Y. Zhu H. Weng M. Chen B. Wang C. Sun L. (2020b). Baohuoside-1 targeting mTOR inducing apoptsis to inhibit hepatocellular carcinoma proliferation, invasion and migration. Biomed. Pharmacother. 128, 110366. 10.1016/j.biopha.2020.110366 32526459

[B21] Hou J. Wang J. Sun E. Yang L. Yan H.-M. Jia X.-B. (2016). Preparation and evaluation of icariside II-loaded binary mixed micelles using Solutol HS15 and Pluronic F127 as carriers. Drug Deliv. 23 (9), 3248–3256. 10.3109/10717544.2016.1167270 26984338

[B22] Huang C. Chen X. Guo B. Huang W. Shen T. Sun X. (2012). Induction of apoptosis by Icariside II through extrinsic and intrinsic signaling pathways in human breast cancer MCF7 cells. Biosci. Biotechnol. Biochem. 76 (7), 1322–1328. 10.1271/bbb.120077 22785466

[B23] Jin X. Zhang Z. H. Sun E. Qian Q. Tan X. B. Jia X. B. (2012). Preparation of a nanoscale baohuoside I-phospholipid complex and determination of its absorption: *in vivo* and *in vitro* evaluations. Int. J. Nanomedicine 7, 4907–4916. 10.2147/IJN.S35965 23028219 PMC3446837

[B24] Jin X. Zhang Z.-H. Sun E. Tan X.-B. Zhu F.-X. Jia X.-B. (2013). A novel drug-phospholipid complex loaded micelle for baohuoside I enhanced oral absorption:in vivoandin vivoevaluations. Drug Development Ind. Pharm. 39 (9), 1421–1430. 10.3109/03639045.2012.719234 23057574

[B25] Kataoka T. (2005). The caspase-8 modulator c-FLIP. Crit. Rev. Immunol. 25 (1), 31–58. 10.1615/critrevimmunol.v25.i1.30 15833082

[B26] Kim B. Park B. (2014). Baohuoside I suppresses invasion of cervical and breast cancer cells through the downregulation of CXCR4 chemokine receptor expression. Biochemistry 53 (48), 7562–7569. 10.1021/bi5011927 25407882

[B27] Kudo-Saito C. Ozaki Y. Imazeki H. Hayashi H. Masuda J. Ozawa H. (2021). Targeting oncoimmune drivers of cancer metastasis. Cancers 13 (3), 554. 10.3390/cancers13030554 33535613 PMC7867187

[B28] Lee K.-S. Lee H.-J. Ahn K. S. Kim S.-H. Nam D. Kim D. K. (2009). Cyclooxygenase-2/prostaglandin E2 pathway mediates icariside II induced apoptosis in human PC-3 prostate cancer cells. Cancer Lett. 280 (1), 93–100. 10.1016/j.canlet.2009.02.024 19289254

[B29] Lee S.-E. Okhlopkova Z. Lim C. Cho S. (2020). Dracocephalum palmatum Stephan extract induces apoptosis in human prostate cancer cells via the caspase-8-mediated extrinsic pathway. Chin. J. Nat. Medicines 18 (10), 793–800. 10.1016/S1875-5364(20)60019-X 33039058

[B30] Li S. Zhan Y. Xie Y. Wang Y. Liu Y. (2020). The impact of icariside II on human prostate cancer cell proliferation, mobility, and autophagy via PI3K-AKT-mTOR signaling pathway. Drug Des. Devel. Ther. Vol.14, 4169–4178. 10.2147/DDDT.S268524 PMC7549881 33116405

[B31] Lim J. Murthy A. (2020). Targeting autophagy to treat cancer: challenges and opportunities. Front. Pharmacol. 11, 590344. 10.3389/fphar.2020.590344 33381037 PMC7768823

[B32] Ma H. Y. Liu X. Z. Liang C. M. (2016). Inflammatory microenvironment contributes to epithelial-mesenchymal transition in gastric cancer. World J. Gastronterol. 22 (29), 6619–6628. 10.3748/wjg.v22.i29.6619 PMC4970470 27547005

[B33] Majidpoor J. Mortezaee K. (2021). Steps in metastasis: an updated review. Med. Oncol. 38 (1), 3. 10.1007/s12032-020-01447-w 33394200

[B34] Mansilla S. F. de la Vega M. B. Calzetta N. L. Siri S. O. Gottifredi V. (2020). CDK-independent and PCNA-dependent functions of p21 in DNA replication. Genes 11 (6), 593. 10.3390/genes11060593 PMC7349641 32481484

[B35] Mercer T. J. Gubas A. Tooze S. A. (2018). A molecular perspective of mammalian autophagosome biogenesis. J. Biol. Chem. 293 (15), 5386–5395. 10.1074/jbc.R117.810366 29371398 PMC5900756

[B36] Nguyen K. T. P. Druhan L. J. Avalos B. R. Zhai L. Rauova L. Nesmelova I. V. (2020). CXCL12-CXCL4 heterodimerization prevents CXCL12-driven breast cancer cell migration. Cell Signal. 66, 109488. 10.1016/j.cellsig.2019.109488 31785332

[B37] Ni F. Tang H. Wang C. Zhang H. Zheng C. Zhang N. (2019). Baohuoside I inhibits the proliferation of pancreatic cancer cells via mTOR/S6K1-caspases/bcl2/bax apoptotic signaling. Cmar Vol. 11, 10609–10621. 10.2147/CMAR.S228926 PMC6927568 31908533

[B38] Peng Y.-G. Zhang L. (2018). Baohuoside-I suppresses cell proliferation and migration by up-regulating miR-144 in melanoma. Pharm. Biol. 56 (1), 43–50. 10.1080/13880209.2017.1418391 29260980 PMC6130571

[B39] Quan K. Zhang X. Fan K. Liu P. Yue Q. Li B. (2017). Icariside II induces cell cycle arrest and apoptosis in human glioblastoma cells through suppressing Akt activation and potentiating FOXO3a activity. Am. J. Transl Res. 9 (5), 2508–2519. 28560001 PMC5446533

[B40] Ribatti D. Vacca A. Presta M. (2000). The discovery of angiogenic factors:. Gen. Pharmacol. Vasc. Syst. 35 (5), 227–231. 10.1016/s0306-3623(01)00112-4 11888677

[B41] Scheau C. Badarau I. A. Costache R. Caruntu C. Mihai G. L. Didilescu A. C. (2019). The role of matrix metalloproteinases in the epithelial-mesenchymal transition of hepatocellular carcinoma. Anal. Cell Pathol. 2019, 1. 10.1155/2019/9423907 PMC6899323 31886121

[B42] Shah A. R. Banerjee R. (2011). Effect of d-α-tocopheryl polyethylene glycol 1000 succinate (TPGS) on surfactant monolayers. Colloids Surf. B: Biointerfaces 85 (2), 116–124. 10.1016/j.colsurfb.2011.01.021 21398100

[B43] Silasi D. A. Alvero A. B. Illuzzi J. Kelly M. Chen R. Fu H. H. (2006). MyD88 predicts chemoresistance to paclitaxel in epithelial ovarian cancer. Yale J. Biol. Med. 79 (3-4), 153–163. 17940625 PMC1994803

[B44] Song J. Feng L. Zhong R. Xia Z. Zhang L. Cui L. (2017). Icariside II inhibits the EMT of NSCLC cells in inflammatory microenvironment via down-regulation of Akt/NF-κB signaling pathway. Mol. Carcinog. 56 (1), 36–48. 10.1002/mc.22471 26859114

[B45] Song J. Shu L. Zhang Z. Tan X. Sun E. Jin X. (2012). Reactive oxygen species-mediated mitochondrial pathway is involved in Baohuoside I-induced apoptosis in human non-small cell lung cancer. Chemico-Biol. Interact. 199 (1), 9–17. 10.1016/j.cbi.2012.05.005 22687635

[B46] Sun Y. S. Thakur K. Hu F. Cespedes-Acuña C. L. Zhang J. G. Wei Z. J. (2020a). Icariside II suppresses cervical cancer cell migration through JNK modulated matrix metalloproteinase-2/9 inhibition *in vitro* and *in vivo* . Biomed. Pharmacother. 125, 110013. 10.1016/j.biopha.2020.110013 32092821

[B47] Sun Y. S. Thakur K. Hu F. Zhang J. G. Wei Z. J. (2020b). Icariside II inhibits tumorigenesis via inhibiting AKT/Cyclin E/CDK 2 pathway and activating mitochondria-dependent pathway. Pharmacol. Res. 152, 104616. 10.1016/j.phrs.2019.104616 31883767

[B48] Teleanu R. I. Chircov C. Grumezescu A. M. Teleanu D. M. (2019). Tumor angiogenesis and anti-angiogenic strategies for cancer treatment. J. Clin. Med. 9 (1), 84. 10.3390/jcm9010084 PMC7020037 31905724

[B49] Thomas M. Davis T. Loos B. Sishi B. Huisamen B. Strijdom H. (2018). Autophagy is essential for the maintenance of amino acids and ATP levels during acute amino acid starvation in MDAMB231 cells. Cell Biochem. Funct. 36 (2), 65–79. 10.1002/cbf.3318 29399832

[B50] Wang L. Lu A. Liu X. Sang M. Shan B. Meng F. (2011). The flavonoid Baohuoside-I inhibits cell growth and downregulates survivin and cyclin D1 expression in esophageal carcinoma via β-catenin-dependent signaling. Oncol. Rep. 26 (5), 1149–1156. 10.3892/or.2011.1400 21785828

[B51] Wu J. Guan M. Wong P. F. Yu H. Dong J. Xu J. (2012). Icariside II potentiates paclitaxel-induced apoptosis in human melanoma A375 cells by inhibiting TLR4 signaling pathway. Food Chem. Toxicol. 50 (9), 3019–3024. 10.1016/j.fct.2012.06.027 22743248

[B52] Wu J. Song T. Liu S. Li X. Li G. Xu J. (2015). Icariside II inhibits cell proliferation and induces cell cycle arrest through the ROS-p38-p53 signaling pathway in A375 human melanoma cells. Mol. Med. Rep. 11 (1), 410–416. 10.3892/mmr.2014.2701 25333296

[B53] Wu J. Zuo F. Du J. Wong P. F. Qin H. Xu J. (2013). Icariside II induces apoptosis via inhibition of the EGFR pathways in A431 human epidermoid carcinoma cells. Mol. Med. Rep. 8 (2), 597–602. 10.3892/mmr.2013.1557 23807305

[B54] Yan H. M. Zhang Z. Jia X. Song J. (2016). D-α-Tocopheryl polyethylene glycol succinate/Solutol HS 15 mixed micelles for the delivery of baohuoside I against non-small-cell lung cancer: optimization and *in vitro*, *in vivo* evaluation. Int. J. Nanomed. Vol. 11, 4563–4571. 10.2147/IJN.S112204 PMC5019457 27660448

[B55] Yan L. Xu F. Dai C.-l. (2018). Relationship between epithelial-to-mesenchymal transition and the inflammatory microenvironment of hepatocellular carcinoma. J. Exp. Clin. Cancer Res. 37 (1), 203. 10.1186/s13046-018-0887-z 30157906 PMC6114477

[B56] Yang D. L. Xin M. M. Wang J. S. Xu H. Y. Huo Q. Tang Z. R. (2015). Chemokine receptor CXCR4 and its ligand CXCL12 expressions and clinical significance in bladder cancer. Genet. Mol. Res. 14 (4), 17699–17707. 10.4238/2015.December.21.43 26782415

[B57] Yun C. W. Jeon J. Go G. Lee J. H. Lee S. H. (2020). The dual role of autophagy in cancer development and a therapeutic strategy for cancer by targeting autophagy. Int. J. Mol. Sci. 22 (1), 179. 10.3390/ijms22010179 PMC7795059 33375363

[B58] Zhang J. Li S. Zhang S. Wang Y. Jin S. Zhao C. (2020). Effect of icariside II and metformin on penile erectile function, histological structure, mitochondrial autophagy, glucose-lipid metabolism, angiotensin II and sex hormone in type 2 diabetic rats with erectile dysfunction. Sex. Med. 8 (2), 168–177. 10.1016/j.esxm.2020.01.006 32147433 PMC7261708

[B59] Zhou J. Wu J. Chen X. Fortenbery N. Eksioglu E. Kodumudi K. N. (2011). Icariin and its derivative, ICT, exert anti-inflammatory, anti-tumor effects, and modulate myeloid derived suppressive cells (MDSCs) functions. Int. Immunopharmacol 11 (7), 890–898. 10.1016/j.intimp.2011.01.007 21244860 PMC3109231

